# Role and regulation of Cdc25A phosphatase in neuron death induced by NGF deprivation or *β*-amyloid

**DOI:** 10.1038/cddiscovery.2016.83

**Published:** 2016-12-12

**Authors:** Nandini Chatterjee, Priyankar Sanphui, Stav Kemeny, Lloyd A Greene, Subhas C Biswas

**Affiliations:** 1 Cell Biology and Physiology Division, CSIR-Indian Institute of Chemical Biology, 4 Raja S. C. Mullick Road, Kolkata 700 032, India; 2 Department of Pathology and Cell Biology, Columbia University Medical Center, New York, NY 10032, USA

## Abstract

Neuron death during development and in Alzheimer’s disease (AD) is associated with aberrant regulation/induction of cell cycle proteins. However, the proximal events in this process are unknown. Cell cycle initiation requires dephosphorylation of cyclin-dependent kinases by cell division cycle 25A (Cdc25A). Here, we show that Cdc25A is essential for neuronal death in response to NGF deprivation or *β*-amyloid (A*β*) treatment and describe the mechanisms by which it is regulated in these paradigms. Cdc25A mRNA, protein and Cdc25A phosphatase activity were induced by NGF deprivation and A*β* treatment. Enhanced Cdc25A expression was also observed in rat brains infused with A*β* and in A*β*-overexpressing A*β*PPswe-PS1dE9 mice. In cultured neurons Cdc25A inhibition by chemical inhibitors or shRNA prevented cell death and neurite degeneration caused by NGF deprivation or A*β*. Additionally, Cdc25A inhibition diminished distal signaling events including Cdk-dependent elevation of phospho-pRb and subsequent caspase-3 activation. Mechanism studies revealed that Cdc25A induction by NGF deprivation and A*β* is mediated by activation of Forkhead transcription factors that in turn suppress miR-21, a negative regulator of Cdc25A. Our studies thus identify Cdc25A as a required upstream element of the apoptotic cell cycle pathway that is required for neuron death in response to trophic factor deprivation and to A*β* exposure and therefore as a potential target to suppress pathologic neuron death.

## Introduction

Neuron death is a physiological process during development and contributes to the pathophysiology of various neurodegenerative diseases including Alzheimer’s disease (AD). During development about half of neurons die due to lack of target-derived trophic support such as caused by limiting supplies of NGF.^1^ In AD, a major cause of neuron degeneration is thought to be due to oligomerization and accumulation of *β*-amyloid (A*β*) protein.^2–6^ Alterations of NGF metabolism and signaling are also implicated in AD.^7,8^ However, the mechanisms of neuron death in the absence of NGF or in response to oligomeric A*β* remain incompletely understood. Aside from increasing understanding of the nervous system, a comprehensive description of neuronal death mechanisms could provide insight to better strategies for treatment of diseases characterized by neuron degeneration.

Accumulating evidence strongly suggests that in response to a wide variety of proapoptotic conditions, including trophic factor deprivation, exposure to A*β*, DNA damage and oxidative stress, postmitotic neurons emerge from the G_0_ state of the cell cycle with aberrant and potentially fatal expression/activation of cell cycle proteins.^9–16^ Studies with NGF deprivation, A*β* treatment or DNA-damaging agents have yielded a consistent set of events related to the cell cycle that culminate in apoptotic neuron death. Among initial responses is activation of G1/S cyclin-dependent kinases (Cdks) such as Cdk4. This in turn phosphorylates retinoblastoma (pRb) family proteins and leads to dissociation of repressor complexes comprising E2F and pRb proteins such as p130, so that E2F-binding genes are de-repressed. Among genes that are de-repressed by loss of E2F–Rb family complexes are the B- and C-myb transcription factors and these in turn transactivate Bim, a proapoptotic protein that promotes caspase activation and subsequent neuron death.^12,13,17,18^

Exit from G_0_/G_1_ and initiation of the cell cycle requires dephosphorylation of inhibitory phosphates on adjacent threonine and tyrosine residues of Cdks such as Cdk4. This is accomplished by the dual specificity phosphatase, cell division cycle 25A (Cdc25A) a member of a phosphatase family comprising Cdc25 A, B and C.^19^

The current work addresses several key unanswered questions regarding the potential role of Cdc25A in neuron death. First, does Cdc25A in particular play a required role in neuron death and activation of the apoptotic cell cycle pathway caused by neurotrophic deprivation? Is this also the case for A*β* treatment? Is Cdc25A upstream of other known events in the pathway? Do neurotrophic deprivation and A*β* treatment lead to elevated Cdc25A levels? If so, what is the signaling mechanism that links induction of Cdc25A to these apoptotic stimuli? Because Cdc25A is an inhibitable enzyme, addressing these issues identifies Cdc25A as a potential target to block pathologic neuron degeneration and death.

## Results

### Early induction and activation of Cdc25A following NGF withdrawal

**Figure 1:**
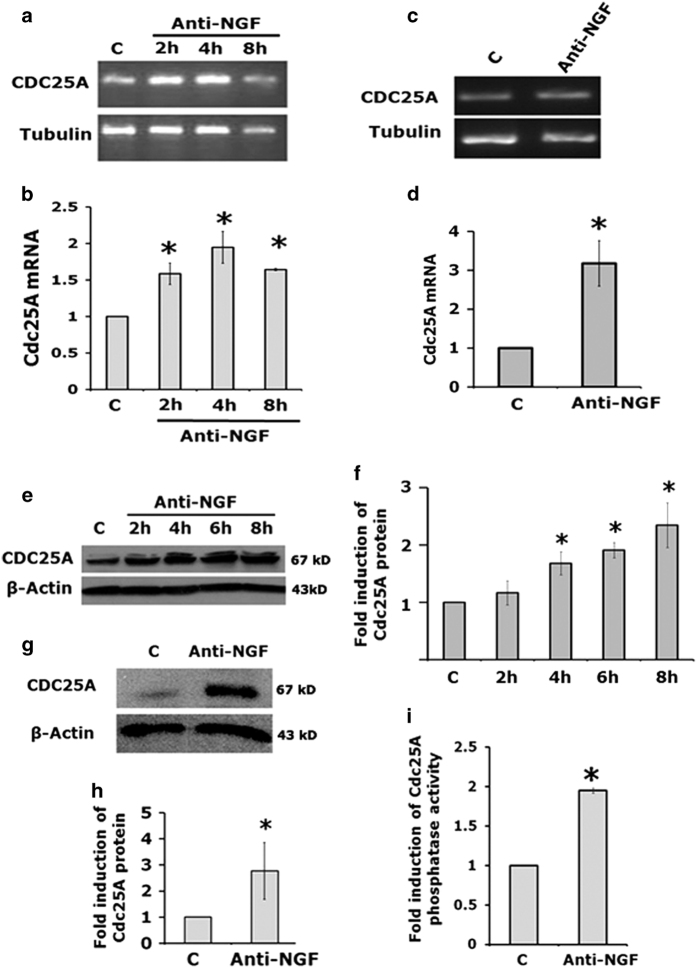
Cdc25A RNA levels, protein levels and its activity are elevated in neuronal cells following NGF deprivation. (**a** and **b**) Neuronally differentiated PC12 cells were subjected to NGF deprivation for indicated times and total RNA was isolated from harvested cells. (**a**) The RNA was analyzed by semiquantitative PCR for Cdc25A transcript levels. *α*-Tubulin was used as a loading control. Representative figure of three independent experiments with similar results is shown. (**b**) Quantitative real-time PCR of Cdc25A transcripts levels. GAPDH was used as a loading control. Data are represented as mean±S.E.M. of four independent experiments. Asterisks denote statistically significant differences from control, *P<0.05. (**c** and **d**) Primary rat sympathetic neurons (5 DIV) were subjected to NGF deprivation for 2 h. Total RNA was isolated from harvested cells. (**c**) The RNA was analyzed by semiquantitative PCR for Cdc25A transcript levels. *α*-Tubulin was used as loading control. A representative image is shown of one of two independent experiments with similar results. (**d**) The RNA was analyzed by quantitative RT-PCR for Cdc25A transcript levels as described in (**b**). Data are represented as mean±S.E.M. of three independent experiments, *P<0.05. (**e**) Neuronally differentiated PC12 cells were treated with anti-NGF for indicated times. Total protein was subjected to western blotting analysis using specific Cdc25A antibody. *β*-Actin was used as a loading control. A representative immunoblot of four independent experiments with similar results is shown. (**f**) Graphical representation of the Cdc25A protein levels as quantified by densitometry of western blots in neuronal PC12 cells subjected to NGF deprivation for 8 h. Data are represented as mean±S.E.M. of four independent experiments, *P<0.05. (**g**) Primary rat sympathetic neurons (5 DIV) were subjected to NGF deprivation for 8 h. Total cell lysates were subjected to western blotting analysis for Cdc25A protein levels. *β*-Actin was used as a loading control. A representative immunoblot of three independent experiments with similar results is shown. (**h**) Graphical representation of the Cdc25A protein levels as quantified by densitometry of western blots in sympathetic neurons subjected to NGF deprivation for 8 h. Data are represented as mean±S.E.M. of three independent experiments, *P<0.05. (**i**) Differentiated PC12 cells were subjected to NGF withdrawal for 8 h. Cdc25A was immunoprecipated and subjected to phosphatase activity assay as described under ‘Materials and Methods’. Data represent mean±S.E.M. of three independent experiments, *P<0.05.

To examine whether Cdc25A plays a role in neuron death, we initially employed neuronally differentiated PC12 cells. PC12 cells neuronally differentiate in the presence of NGF and require NGF for survival in the absence of serum.^20^ Like sympathetic neurons, upon NGF deprivation these cells undergo apoptotic death starting at about 16 h with about half dying by 24 h.^21^ Assessment of Cdc25A transcripts levels in neuronal PC12 cells following NGF withdrawal by both semiquantitative (Figure 1a) and quantitative RT-PCR (Figure 1b) revealed significantly increased Cdc25A mRNA levels within 2 h of NGF withdrawal. We confirmed these results in primary cultures of rat neonatal sympathetic neurons cultured for 5 days and subjected to NGF deprivation for 2 h. In this case also, semiquantitative (Figure 1c) and quantitative PCR (Figure 1d) showed significantly increase in Cdc25A transcripts following 2 h of NGF withdrawal.

We next determined whether the increase in Cdc25A transcripts was reflected in Cdc25A protein levels. Western blotting showed that Cdc25A protein levels significantly increased in a time-dependent manner by 2–3-fold in neuronal PC12 cells following NGF withdrawal (Figures 1e and f) and in primary sympathetic neurons following 8 h of NGF withdrawal (Figures 1g and h).

We also assessed whether Cdc25A phosphatase activity is similarly increased in response to 8 h NGF deprivation and observed a significant doubling of activity compared with that of controls (Figure 1i). Collectively, these experiments indicate that NGF deprivation causes a relatively rapid induction of Cdc25A mRNA, protein and activity well before death is evident.

### Cdc25A activity and expression are required for death caused by NGF deprivation

**Figure 2:**
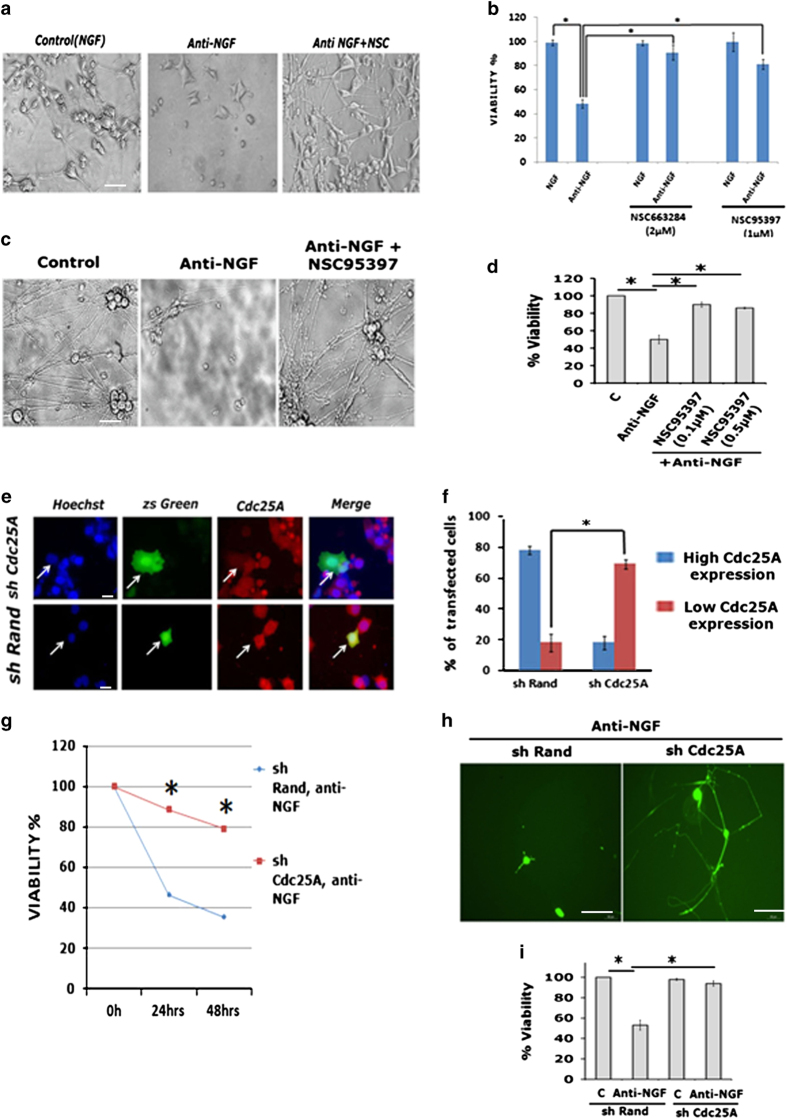
Blocking Cdc25A by either pharmacological inhibitors or shRNA protects neuronal PC12 cells and primary sympathetic neurons from death induced by NGF deprivation. (**a**) Neuronal PC12 cells (5 DIV) were subjected to NGF deprivation in the presence and absence of NSC663284 or NSC95397 at the indicated concentrations for 20 h. Upper panel: representative phase contrast micrographs show retention of neurites after NGF deprivation in the presence of NSC663284 (2 *μ*M). Scale bar: 50 *μ*M. (**b**) Graphical representation of relative percentage of viable cells under the indicated conditions. Data are represented as mean±S.E.M. of three independent experiments performed in duplicates. The asterisk denotes statistically significant difference between indicated classes: *P<0.01. (**c** and **d**) Primary sympathetic neurons (5 DIV) were exposed to anti-NGF for overnight in the presence or absence of Cdc25 inhibitor NSC95397 at two different concentrations (0.1 and 0.5 *μ*M). (**c**) Representative phase contrast micrographs of primary sympathetic cultures under the indicated conditions. Scale bar: 50 *μ*M. (**d**) The corresponding quantitative data derived from direct counting of viable nuclei. Data are represented as mean±S.E.M. of three experiments. Asterisks denote statistically significant differences between the indicated groups. *P<0.005. (**e** and **f**) shRNA targeted to Cdc25A blocks expression of endogenous Cdc25A. (**e**) Undifferentiated PC12 cells that were transfected with the indicated constructs, maintained for 48 h and then immunostained with antibody against Cdc25A. Scale bar: 10 *μ*M. (**f**) The percentage of transfected cells shows the proportion of transfected cells with high (more or equal than the neighboring non-transfected cells) or low (less than the neighboring non-transfected cells) Cdc25A immunoreactivity levels. Data are represented as mean±S.D. of two experiments: *P<0.01. (**g**) Differentiated PC12 cells (5 DIV) were transfected with shRand-zsgreen or shCdc25A-zsgreen and 48 h post transfection, the cells were subjected to NGF deprivation. The numbers of surviving-transfected (green) cells were counted at the indicated times under fluorescence microscopy. The percentages of viable cells are represented graphically. Data are represented as mean±S.D. of two independent experiments. The asterisk denotes statistically significant difference between indicated class: *P<0.05. (**h** and **i**) Primary rat sympathetic neurons (5 DIV) were transfected with shRand-zsgreen or shCdc25A-zsgreen. Forty-eight hours post transfection, cells were subjected to overnight NGF deprivation. The numbers of surviving-transfected (green) cells were counted under fluorescence microscopy. (**h**) Representative images of transfected sympathetic neurons after NGF deprivation under each condition. Scale bar: 100 *μ*M. (**i**) Graphical representation of percentage of viable cells. Data are represented as mean±S.E.M. of three experiments. Asterisks denote statistically significant difference between the indicated groups. *P<0.05.

Next we investigated whether Cdc25A activity and expression are required for NGF deprivation-induced cell death. To assess the activity requirement, we treated neuronal PC12 cells with anti-NGF in the presence and absence of the Cdc25 inhibitors NSC663284 and NSC95397 and assayed cell viability. Both drugs conferred protection and, importantly, protected neurites from degeneration (Figures 2a and b). A similar experiment with rat sympathetic neuron cultures revealed that NSC95397 showed significant protection from NGF deprivation (Figures 2c and d) as well as neurite retention (Figure 2c).

Because inhibitors may have nonspecific effects and are not selective (that is also block Cdc25B and C), we used shRNA to specifically suppress Cdc25A expression. Efficacy of the construct was verified in that it substantially diminished expression of endogenous and exogenously expressed Cdc25A (Figures 2e and f and data not shown). Compared with a control construct (shRand, a scrambled shRNA), shCdc25A significantly protected neuronal PC12 cells and sympathetic neurons from death induced by NGF deprivation (Figures 2g–i). Moreover, a majority of shCdc25A-transfected neurons maintained their overall neuronal morphology after NGF deprivation (Figure 2h). Taken together these experiments indicate that interfering with Cdc25A activity or expression not only protects neurons from death but also preserves their morphology following NGF deprivation. Thus, Cdc25A is a required element in the death mechanism.

### Cdc25A is induced in cortical neurons by A*β*

**Figure 3:**
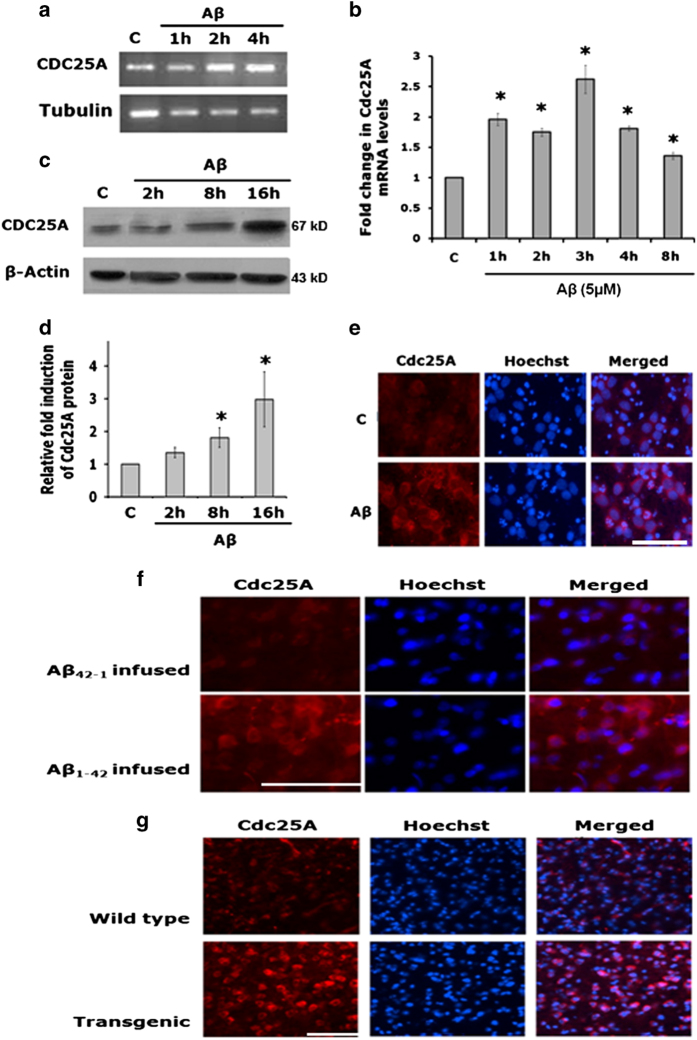
Cdc25A levels are elevated in primary rat cortical neurons and *in vivo* in response to A*β*. (**a**) Primary rat cortical neurons were treated with A*β* for indicated times. Total RNA was isolated and used for semiquantitative PCR with specific primers for Cdc25A and *α*-tubulin. PCR products were resolved in a 1.5% agarose gel and stained with ethidium bromide. A representative gel image shows the relative Cdc25A and tubulin mRNA levels. (**b**) Neuronally differentiated PC12 cells were treated with A*β* for indicated times. Total RNA was isolated and used for quantitative PCR with specific primers for Cdc25A. GAPDH was used as a loading control. The image shows graphical representation of mRNA of Cdc25A following A*β* treatment. (**c**) Primary rat cortical neurons were treated with A*β* for indicated times. Total proteins were extracted from A*β* -treated cortical neurons and analyzed by western blotting analysis for Cdc25A protein levels. A representative immunoblot of three independent experiments with similar results is shown. (**d**) Graphical representation of Cdc25A protein levels as quantified by densitometry. Data are represented as mean±S.E.M. of three independent experiments. Asterisks denote statistically significant differences between the indicated groups. *P<0.05. (**e**) Primary rat cortical neurons were treated with A*β* for 16 h and immunostained with Cdc25A antibody. Hoechst dye 33342 was used to stain the nuclei. Representative images from one of three independent experiments with similar results are shown. Scale bar: 100 *μ*M. (**f**) Oligomeric A*β*_(1–42)_ was infused into rat brain as described under ‘Materials and Methods’. The animals were maintained for 21 days before killing. Brain sections were immunostained for Cdc25A. The reverse peptide A*β*_(42–1)_ was infused into control animals. Nuclei were stained with Hoechst dye 3342. Representative images of two sections from two different animals for each group with similar results are shown. Scale bar: 100 *μ*M. (**g**) Brain sections of wild type and transgenic (A*β*PPswe-PS1dE9) mice were immunostained with anti-Cdc25A antibody and nuclei were stained with Hoechst dye 3342. Representative images of two sections from three different transgenic animals and two wild-type animals with similar results are shown. Scale bar: 100 *μ*M.

An extensive literature links NGF, AD and A*β*^22^ and the molecular mechanisms of neuronal cell death evoked by either NGF deprivation or A*β* treatment have many commonalities including activation of cell cycle molecules.^13^ We therefore examined whether Cdc25A is affected and plays a role in A*β*-induced neurodegeneration. Cultured cortical neurons and neuronal PC12 cells were exposed to 1.5 *μ*M oligomeric A*β* and 5 *μ*M oligomeric A*β,* respectively (Figures 3a and b), levels that cause cell death within 24 h.^23^ Semiquantitative PCR showed a doubling in Cdc25A transcripts within 2 h of treatment in cortical cultures (Figure 3a) and a similar change within an hour in PC12 cell cultures (Figure 3b). The increase in transcripts in cortical neurons was reflected by a significant two- to three-fold elevation of Cdc25A protein expression by 8–16 h of A*β* exposure (Figures 3c and d). Immunocytochemical analysis also confirmed increased Cdc25A protein levels in cortical neurons following A*β* treatment (Figure 3e). Our results thus indicate that Cdc25A is elevated in neurons by A*β* and by NGF deprivation and that this occurs well before onset of death.

### Cdc25A is elevated *in vivo* in AD models

To complement our culture studies, we next asked whether Cdc25A is induced in *in vivo* models of AD. To do so, we infused oligomeric A*β* or the reverse peptide A*β*_(42–1)_ into right hemispheres of rat brains. We and others have shown that this causes A*β* accumulation and neuron death near the infusion site.^23^ The animals were killed 21 days post infusion. Immunohistochemical analysis of the brains revealed a significant upregulation of Cdc25A protein levels near the sites of infusion with A*β* compared with infusion with A*β*_(42–1)_ (Figure 3f).

As a second model, we examined brain sections of a*β*PPswe-PS1sde9 (Swedish mutations in APP and PS1) transgenic mice for Cdc25A expression. Congo red staining confirmed significant amounts of A*β* plaques in the transgenic mouse brains (data not shown), with no such plaques in control littermates. Immunohistochemical analysis showed a considerable increase in Cdc25A expression in the transgenic mice compared with control littermates (Figure 3g). Collectively, these findings indicate that Cdc25A is upregulated in both *in vitro* and *in vivo* models of AD.

### Cdc25A is required for A*β*-induced neuron death and degeneration

**Figure 4:**
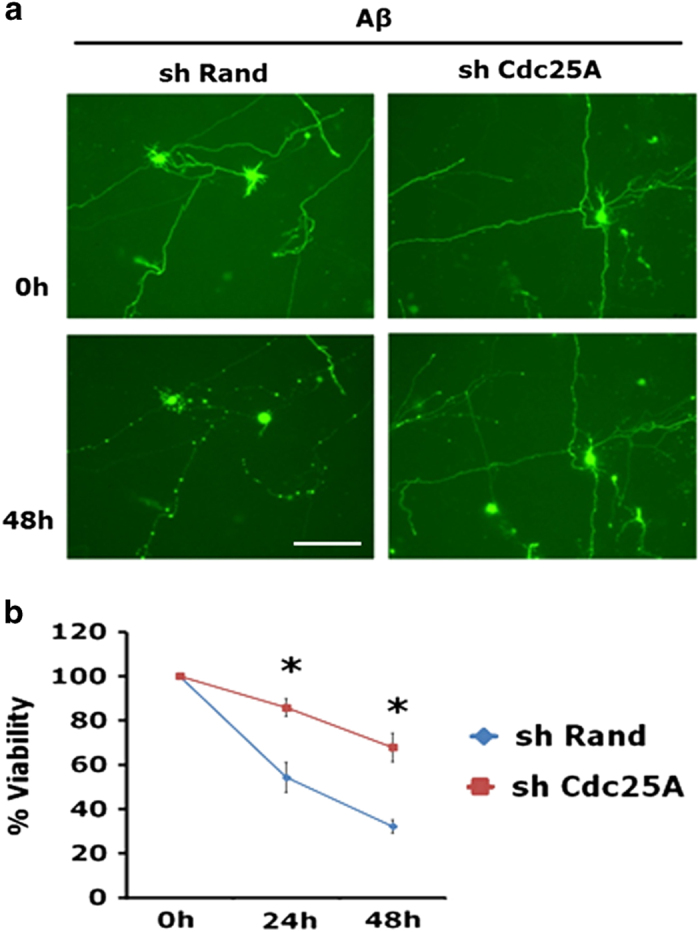
siRNA-mediated knockdown of Cdc25A protects primary cortical neurons against A*β* toxicity. Primary rat cortical neurons (7 DIV) were transfected with shRand-zsgreen or shCdc25A-zsgreen. Forty-eight hours post transfection, neurons were treated with A*β*. Live green cells were counted at the indicated times after A*β* treatment under fluorescence microscopy. (**a**) Representative images of cortical neurons transfected with shRand-zsgreen or shCdc25A-zsgreen and then exposed to A*β* for 48 h. (**b**) Graphical representation of the percentage of viable cells under each condition. Data are represented as mean±S.E.M. of three independent experiments performed in duplicates. The asterisks denote statistically significant difference from control (shRand): *P<0.005. Scale bar: 100 *μ*M.

We next examined whether Cdc25A is required in A*β*-induced neuronal death as it is for NGF withdrawal. Cortical neuron cultures were transfected with shCdc25A or shRand and exposed to A*β* and living transfected (Zsgreen+) cells were counted at 24 and 48 h. Cdc25A downregulation provided significant protection to neurons from death evoked by A*β* (Figure 4a and b). Moreover, Cdc25A knocked down neurons retained normal neuronal morphology after A*β* exposure, which contrasted with degeneration of neurites in A*β*-treated shRand-transfected neurons (Figure 4a).

### Cdc25A lies upstream of and is required for Rb phosphorylation and caspase-3 cleavage after NGF deprivation or A*β* treatment

**Figure 5:**
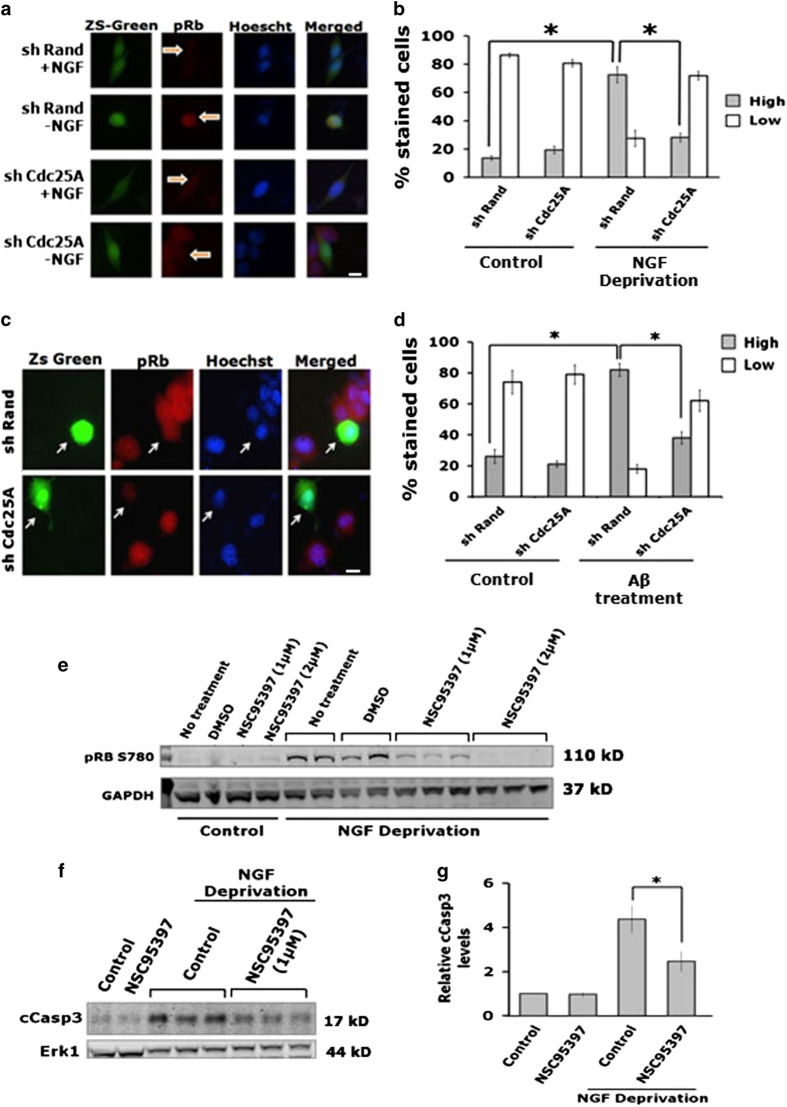
Silencing of Cdc25A by an shRNA successfully blocks phosphorylation of Rb protein in response to NGF deprivation or A*β* in neuronal PC12 cells. (**a**) Neuronal PC12 cells were transfected with the indicated constructs, maintained for 48 h and then subjected to NGF deprivation for 20 h. Following NGF deprivation, cells were immunostained with antibody against phospho-Rb. Representative images from one of two different experiments with similar results are shown. Scale bar: 10 *μ*M. (**b**) The percentage of transfected cells with high (more or equal than the neighboring non-transfected cells) or low (less than the neighboring non-transfected cells) phospho-Rb immunoreactivity levels in the presence and absence of NGF are shown. Data are represented as mean±S.D. of two experiments in duplicate. Asterisks denote statistically significant differences between indicated classes, *P<0.01. (**c**) Neuronally differentiated PC12 cells were transfected with the indicated constructs. Forty-eight hours post transfection cells were exposed overnight to A*β* and immunostained for phospho-Rb. Representative images from one of three different experiments with similar results are shown. Scale bar: 10 *μ*M. (**d**) The percentage of transfected cells with high (more or equal than the neighboring non-transfected cells) or low (less than the neighboring non-transfected cells) phospho-Rb immunoreactivity levels in control and A*β*-treated cells are shown. Data are represented as mean±S.D. of two experiments in duplicate. Asterisks denote statistically significant differences between indicated classes, *P<0.01. (**e**) Neuronally differentiated PC12 cells were deprived of NGF for 48 h in the presence and absence of 1  and 2 *μ*M of NSC95397. Total protein was subjected to western blot analysis using phospho-Rb antibody that specifically detects the Serine-780 phosphorylated form of the protein, which is phosphorylated by Cdk4. GAPDH was used as a loading control. (**f**) Neuronally differentiated PC12 cells were treated with anti-NGF in the presence and absence of 1 *μ*M of NSC95397 for 40 h. Total protein was subjected to western blot analysis using specific cleaved Caspase-3 antibody. ERK1 was used as a loading control. A representative immunoblot of four independent experiments with similar results is shown. (**g**) Graphical representation of the cleaved caspase-3 protein levels as quantified by densitometry. Data are represented as mean±S.E.M. of four independent experiments, *P<0.05.

A molecular pathway has been described that includes cell cycle-related molecules and that plays a required role in neuron death induced by NGF deprivation or A*β* treatment.^13^ As dissected thus far, the pathway starts with activation of Cdk4, which in turn phosphorylates proteins of the Rb family, thereby triggering a series of events that lead to caspase-3 activation and subsequent neuron death. We therefore asked whether Cdc25A is upstream of Cdk4 in this scheme. If Cdc25A acts upstream of Cdk4, then suppression/inhibition of Cdc25A should in turn block Cdk4-dependent Rb phosphorylation and subsequent activation of caspase-3 by NGF deprivation or A*β* treatment. Indeed, when we assessed NGF-deprived PC12 cells by immunostaining, we found that shCdc25A treatment greatly reduced the proportion with high levels of phospho-Rb (Figure 5a and b). Similarly, A*β* treatment elevated levels of phosphorylated Rb in neuronal PC12 cells and this was blocked by Cdc25A knockdown (Figure 5c and d). We additionally analyzed phosphorylated Rb by western blotting after Cdc25 inhibition with NSC95397 in the presence and absence of NGF. Blocking Cdc25 activity substantially suppressed phosphorylation induced by NGF deprivation (Figure 5e). Finally, we found that Cdc25 inhibition by NSC95397 significantly blocked NGF-deprivation-induced caspase-3 cleavage (Figure 5f and g). Thus these experiments indicate that Cdc25A is upstream of and required for activation of cell-cycle-related pathways involved in neuronal death following NGF deprivation and A*β* exposure.

### FoxO transcription factors are required for induction of Cdc25A following A*β* treatment

**Figure 6:**
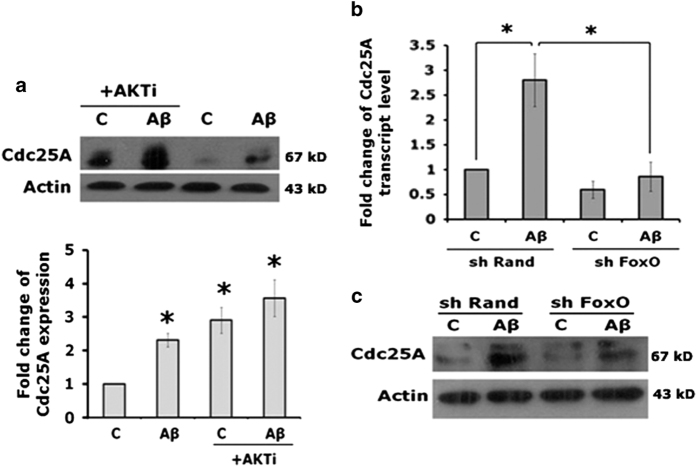
Downregulation of FoxO by shRNA blocks upregulation of Cdc25A in response to A*β* treatment. (**a**) Upper panel: PC12 cells were neuronally differentiated and treated for 16 h with A*β* in the presence or absence of the AKT inhibitor, AKTi. Cells were lysed and total protein was analyzed by western blotting analysis for Cdc25A and actin (loading control). Lower panel: graphical representation of the Cdc25A protein levels as quantified by densitometry. Data are represented as mean±S.E.M. of three independent experiments. Asterisks denote statistically significant differences between the indicated groups. *P<0.05. (**b**) PC12 cells were transfected with shRand or shFoxO and then differentiated in the presence of NGF for 5 days and then treated with A*β* for 4 h. Total RNA was isolated from harvested cells and analyzed by qRT-PCR for Cdc25A transcript levels. GAPDH was used as a loading control. Data are represented as mean±S.D. of two independent experiments, *P<0.05. (**c**) PC12 cells were transfected with shRand or shFoxO, differentiated in the presence of NGF for 5 days and then treated overnight with A*β*. Cells were lysed and total protein was analyzed by western blotting for expression of Cdc25A and actin (loading control). A representative image from one of two experiments with similar results is shown.

Next we explored the molecular mechanism by which Cdc25A expression is regulated. Because Cdc25A is regulated by multiple signaling pathways, including the JNK, ERK and AKT pathways in proliferating cells,^19^ we first assessed specific pathway inhibitors for their effects on Cdc25A expression in neuronal PC12 cells. Although JNK and ERK pathway inhibitors had no effects on Cdc25A levels, inhibition of the AKT pathway for 16 h led to elevated Cdc25A expression comparable to that achieved by exposure to A*β* or NGF deprivation (Figure 6a). FoxO family transcription factors are direct targets of the PI3K/AKT pathway that are inactivated by Akt-mediated phosphorylation and that are thus activated when PI3K/AKT signaling is blocked.^24,25^ We previously reported that FoxO3a activation plays a necessary role in A*β*-induced neurodegeneration^23^ and FoxO activation is required for neuron death caused by NGF deprivation and consequent shut-down of Akt activity.^26,27^ We therefore hypothesized that Cdc25A upregulation in our death models is mediated by FoxOs. To test this in the A*β* model, we knocked down FoxOs in neuronally differentiated PC12 cells with a previously reported shRNA that targets all FoxO isoforms^18^ and determined levels of both Cdc25A transcripts and protein. A*β* upregulated Cdc25A in control (shRand transfected) cells as expected, and this was blocked/diminished in cells in which FoxOs were downregulated by shRNA (Figures 6b and c). Collectively, these results indicate that FoxO transcription factors are required for Cdc25A upregulation by A*β*.

### FoxOs elevate Cdc25A expression by suppressing miR-21 levels following A*β* treatment

**Figure 7:**
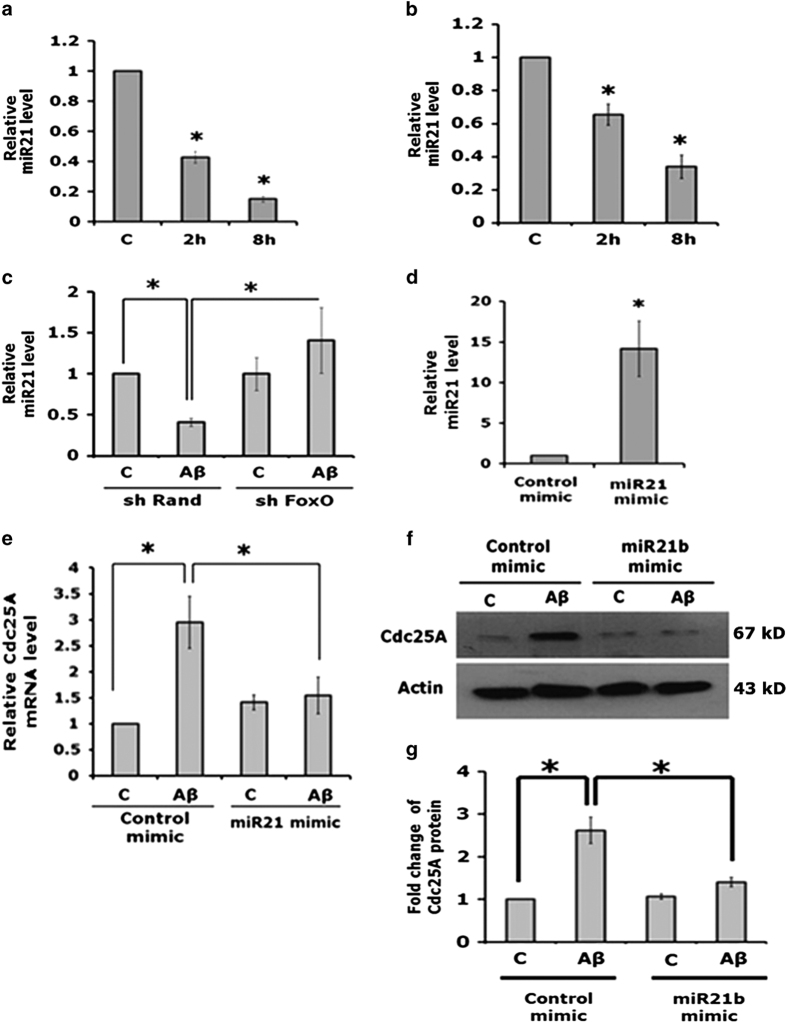
FoxO upregulates Cdc25A by suppressing miR-21 expression following A*β* treatment. (**a**) Cultured cortical neurons (7 DIV) were treated with A*β* for the indicated times. Total RNA was isolated and analyzed by qRT-PCR using the Taqman assay to quantify the relative miR-21 levels. Data are represented as mean±S.D. of two independent experiments, *P<0.05. (**b**) Cultured hippocampal neurons (21 DIV) were treated with A*β* for the indicated times. Total RNA was isolated and analyzed by qRT-PCR using the Taqman assay to quantify the relative miR-21 levels. Data are represented as mean±S.D. of two independent experiments, *P<0.05. (**c**) PC12 cells were transfected with shRand or shFoxO and then differentiated in the presence of NGF for 5 days. Cultures were treated with A*β* for 4 h and relative levels of miR-21 were determined by real-time PCR using the Taqman assay. Data are represented as mean±S.D. of two independent experiments, *P<0.05. (**d)** PC12 cells were transfected with control mimic or miR-21 mimic and differentiated in the presence of NGF for 5 days. Total RNA was isolated and analyzed by qRT-PCR using the Taqman assay for relative levels of miR-21 signal. (**e**) Neuronal PC12 cells (transfected with control or miR-21 mimic) were treated for 4 h with A*β*. Total RNA was isolated and analyzed by qRT-PCR for relative Cdc25A levels. Data are represented as mean±S.D. of two independent experiments, *P<0.05. (**f**) Total lysates from cells treated with A*β* for 16 h were analyzed by western blotting for Cdc25A and actin protein levels. Representative images of one of two experiments with similar results are shown here. (**g**) Graphical representation of the Cdc25A protein levels as quantified by densitometry of western blots in neuronal PC12 cells transfected with control or miR-21 mimic maintained in the presence or absence of A*β*. Data are represented as mean±S.D. of two independent experiments, *P<0.05.

We next probed the mechanism by which FoxOs regulate Cdc25A in response to A*β* treatment. Because FoxOs are transcription factors, we initially considered that they may regulate Cdc25A by binding directly to its promoter. However, examination of the promoter sequence did not reveal potential FoxO binding sites. It has been reported that FoxO3a transcriptionally represses miR-21 in cancer cells following doxorubicin treatment^28^ and that miR-21 negatively regulates Cdc25A.^29^ We therefore assessed whether FoxOs regulate Cdc25A via miR-21 in the context of A*β* treatment. We initially measured miR-21 levels in cortical and hippocampal neurons following A*β* treatment and found a significant reduction at 2 (30–60%) and 8 h (60–90%) after A*β* exposure (Figures 7a and b). To determine whether this response is mediated by FoxOs, we knocked down FoxOs in neuronal PC12 cells, treated them with A*β* and assessed miR-21 levels by RT-PCR. This also revealed a substantial reduction in miR-21 following A*β* treatment and this effect was completely abolished in FoxO knockdown cells (Figure 7c). These findings thus indicate that A*β* negatively regulates miR-21 in neuronal cells and that this action is mediated by FoxO transcription factors.

Next, we evaluated whether the A*β*-promoted decrease in miR-21 levels is responsible for elevating Cdc25A expression. For these experiments, we overexpressed miR-21 activity in neuronally differentiated PC12 cells by transfecting them with an miR-21 mimic or with a control mimic in the presence or absence of A*β*. Our reasoning was that the overexpressed miR-21 mimic should over-ride the inhibitory actions of A*β* on endogenous miR-21 levels and therefore suppress the elevation of Cdc25A caused by A*β* exposure. RT-PCR verified that the cells transfected with the miR-21 mimic showed a large increase in miR-21 signal compared with cells transfected with the control mimic (Figure 7d). RT-PCR also showed upregulation of Cdc25A transcripts as anticipated following A*β* exposure in the presence of the control mimic. Significantly, this upregulation was nearly completely blocked in miR-21 mimic-overexpressing cells (Figure 7e). Western blots also verified upregulation of Cdc25A protein following A*β* treatment in the presence of the control mimic and that this effect was fully suppressed in cells transfected with the miR-21 mimic (Figures 7f and g). Taken together our findings thus support a neuronal pathway in which A*β* treatment activates FoxO transcription factors that in turn downregulate miR-21, leading to elevated Cdc25A expression.

## Discussion

One of the major signaling mechanisms that promote apoptotic neuron death is inappropriate activation of an apoptotic cell cycle pathway. A wealth of data indicates that activation of cell cycle molecules is linked to the core cell death machinery during development and in various neurological conditions.^9,12–14,16,30,31^ In response to apoptotic stimuli, G1/S cyclin-dependent kinases such as Cdk4 are activated and phosphorylate proteins of the pRb family.^32^ This in turn causes dissociation of repressor complexes comprising E2F family members and pRb proteins such as p130.^12,13,33^ Free E2F transcription factors activate myb transcription factors that participate in activation of proapoptotic proteins such as Bim that promote caspase activation and execution of neuron death.^12,13,17,18,33^ However, how Cdk4 is activated in the first place in postmitotic neurons in response to apoptotic stimuli has remained elusive.

One of the mechanisms by which Cdk4 is activated in proliferating cells is by dephosphorylation of its inhibitory phosphates on adjacent threonine and tyrosine residues by the dual specificity phosphatase Cdc25A.^19^ In this study, we identified Cdc25A as a regulator of Cdk4 activity in neuron death in response to NGF deprivation and A*β* treatment. We found that Cdc25A is induced *in vitro* by NGF deprivation and *in vitro* and *in vivo* by A*β* in various relevant models of AD. Moreover, its phosphatase activity is also enhanced to a similar degree in NGF-deprived neuronal cells. Importantly, inhibition of Cdc25A activity/expression by inhibitors or shRNA protected neurons from NGF deprivation and A*β* treatment. These treatments also preserved neuronal morphology including neurites, indicating that Cdc25A and the apoptotic cell cycle pathway are involved in the neurite degeneration associated with trophic factor deprivation and A*β* exposure. Altogether, our observations indicate that Cdc25A is rapidly elevated, activated and plays a required role in developmental and AD-relevant models of neuron death.

Our findings indicate that NGF deprivation and A*β* lead to a rapid two- to three-fold induction of Cdc25A mRNA and protein. NGF withdrawal causes a similar increase in Cdc25A activity. These events occur at about the same time that apoptotic insults lead to Cdk4 activation and Rb phosphorylation in our systems^32^ and well precede evident signs of neuron death. In line with a causal role for Cdc25A in these events, shRNA or an inhibitor targeting Cdc25 diminished phospho-pRb levels induced by NGF deprivation and A*β* treatment. While our findings establish a role for Cdc25A in two death paradigms studied here, they do not indicate whether the increase in expression *per se* is essential for cell death. This possibility is supported by findings that Cdc25A overexpression is sufficient to cause neuronal cell death,^34^ that Cdc25A levels and activity increase in parallel in our study, and that death is averted when Cdc25A levels are reduced by shRNA. In non-neuronal cells, Cdc25A levels are subject to regulation by protein stabilization.^35^ Our observations that Cdc25A transcripts and protein are induced to a similar degree suggest that this is not the case in our paradigms. A variety of post-translational modifications also have been shown to participate in Cdc25A regulation^19,36^ and it is plausible that these too contribute to elevation of neuronal Cdc25A activity in response to apoptotic insults.

Our findings establish for the first time that Cdc25A is a required upstream activator of the apoptotic cell cycle pathway in trophic factor-deprived neurons and that its levels under such conditions are elevated by a pathway involving FoxOs and miR-21. In the case of A*β* there have been both relevant and conflicting findings that are addressed and significantly extended by our study. It has been reported that neurons in post-mortem brains from AD patients have elevated Cdc25A immunostaining and that brain tissue from AD patients has higher Cdc25A phosphatase activity compared with non-AD brains.^37^ Chang *et al.*^38^ found that A*β* activates Cdc25A, B and C in cultured neurons and that a broad-spectrum Cdc25 inhibitor protects from A*β*-induced death. Elevated Cdc25 activity was associated with activation of, and phosphorylation by, cyclin-dependent kinase-5 (Cdk5) with no change in Cdc25 levels. In contrast, Kruman *et al.*^39^ described a three- to four-fold increase in Cdc25A protein in A*β*-treated cortical neuron cultures. Our study thus favors a mechanism in which A*β* elevates Cdc25A expression and activity and provides an explanation for how this occurs via FoxO–miR-21 signaling. Our causal data also clearly identify Cdc25A for the first time as a required player in A*β*-induced neuron death.

Zhang *et al.*^34^ examined Cdc25A in the context of camptothecin-induced DNA damage to cultured neurons. Camptothecin activated Cdc25A within 2 h and inhibition or knockdown of Cdc25A was protective and blocked camptothecin-induced Cdk4 activation and Rb phosphorylation, thus linking it to the cell cycle pathway described above. However, in contrast to NGF deprivation and A*β* treatment, Cdc25A activation by camptothecin did not change Cdc25A levels (and therefore not likely the FoxO–miR-21 pathway), but rather was correlated with loss of activity of the checkpoint 1 kinase (Chk1). Thus, it appears that although distal elements of the neuronal apoptotic cell cycle pathway are similar for different death inducers, multiple mechanisms exist to initiate the pathway via Cdc25A.

**Figure 8:**
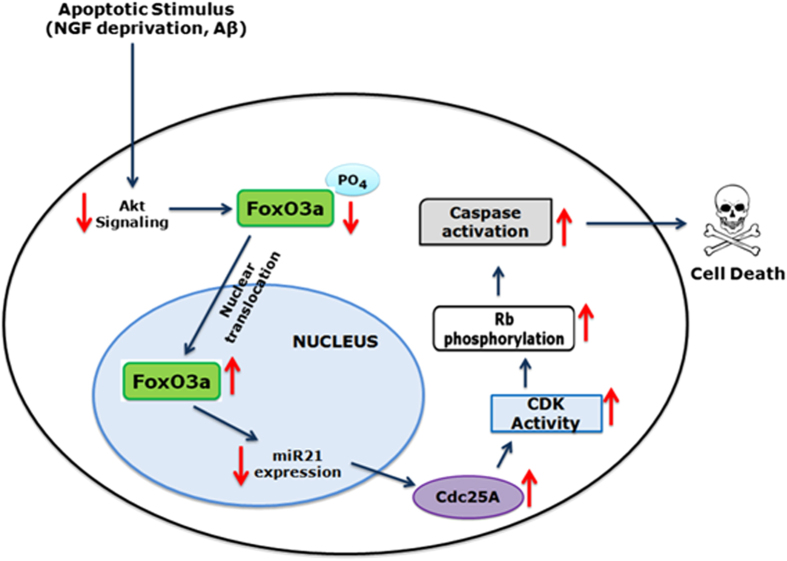
Schematic representation of the mechanism and consequences of Cdc25A induction in response to NGF deprivation or A*β* treatment.

Our investigation of the mechanism of Cdc25A induction in neurons revealed a two- to three-fold elevation when Akt is inhibited. Neuronal Akt signaling is inhibited by oligomeric A*β* ^23^ and Akt activity is diminished in brains of AD patients and of APP trangenic mice.^40^ NGF deprivation also rapidly decreases Akt phosphorylation/activity.^27^ FoxO transcription factors are well-described Akt targets that are activated when Akt signaling is suppressed,^41^ and FoxO activation occurs and is required for neuronal death induced by trophic factor deprivation and A*β* exposure.^18,23,26,27^ These considerations suggested that Cdc25A might be regulated by FoxOs. Indeed, FoxO downregulation by shRNA blocked upregulation of Cdc25A in A*β*-treated neurons. Because the Cdc25A gene has no consensus sequence for binding of FoxO transcription factors, we speculated that it might be regulated post-transcriptionally. It was reported that Cdc25A is suppressed by miR-21 in colon cancer cells^29^ and that FoxO3a induces apoptosis of lung cancer cells by negatively regulating miR-21 in response to doxorubicin.^28^ We found that a miR-21 mimic is sufficient to block Cdc25A mRNA and protein induction by A*β* and that miR-21 is downregulated in A*β*-treated neurons by a mechanism requiring FoxO transcription factors. These findings suggest a pathway in which NGF deprivation or A*β* treatment leads successively to Akt inactivation, FoxO activation, suppression of miR-21 levels and consequent elevation of Cdc25A (Figure 8). Thus, our findings not only place elevated Cdc25A activity upstream of Cdk4 activation in the apoptotic neuronal death pathway, but also indicate a set of signaling events by which loss of trophic support or A*β* exposure may regulate cellular Cdc25A levels and activity.

In conclusion, our study reveals that Cdc25A is both elevated and activated in neuronal cells by apoptotic stimuli relevant to normal development and to AD, and that it plays an essential role in neuronal degeneration and death in both instances. Targeting Cdc25A may therefore be a useful strategy for providing neuroprotection in AD and other pathologies in which the neuronal apoptotic cell cycle pathway is activated. In this regard, a selective Cdc25A inhibitor has been effective in several non-neuronal experimental disease models and without reported toxicity.^42–44^

## Materials and Methods

### Materials

Insulin, progesterone, putrescine, selenium, transferrin, anti-NGF, NGF, poly-d-lysine, NSC663284 and NSC95397 were purchased from Sigma (St Louis, MO, USA). Cell culture media DMEM, DMEM-F12, RPMI-1640, Neurobasal, B27, antibiotics, Lipofectamine 2000, Alexa Fluor, cDNA synthesis kit, real-time PCR kit and serum were purchased from Invitrogen (Life technologies, Grand Island, NY, USA). Cell culture dishes, plates and flasks were purchased from BD Falcon, Corning (Corning, NY, USA). A*β*_(1–42)_ and A*β*_(42-1)_ were purchased from American Peptide (Sunnyvale, CA, USA). ECL reagent and PVDF membrane were purchased from GE Healthcare (Buckinghamshire, UK). The PCR kits were purchased from Takara (Shiga, Japan), Fermentas (Waltham, MA, USA). Anti-Cdc25A, anti-Actin and HRP-conjugated secondary antibodies were purchased from Santa Cruz Biotechnology (Dallas, TX, USA). Anti-phospho-Rb antibody was purchased from Cell Signaling Technologies (Denver, MA, USA). AKT inhibitor II, JNK inhibitor II and U0126 were purchased from Calbiochem (Darmstadt, Germany). Primers were purchased from IDT DNA (Gurgaon, Haryana, India). Brain tissues of A*β*PPswe-PS1de9 mice and control littermates were a kind gift from Dr. Anant B. Patel (Council of Scientific and Industrial Research-Centre for Cellular and Molecular Biology (CSIR- CCMB)), Hyderabad, India.

### Cell culture

Rat pheochromocytoma cells (PC12) cells were cultured as described previously^20^ and were grown in either DMEM or RPMI-1640 medium supplemented with 10% heat inactivated horse serum (HS) and 5% heat inactivated fetal bovine serum. Cells were neuronally differentiated by treatment with NGF (50 ng/ml) for 5–7 days in medium supplemented with 1% HS. Primary cortical neurons were cultured as described.^23,32,45^ In brief, the neocortices of E-18 rat embryo were dissected out, dissociated and cultured in serum-free medium (DMEM/F12 (1:1)) supplemented with 6 mg/ml d-glucose, 100 *μ*g/ml transferrin, 25 *μ*g/ml insulin, 20 nM progesterone, 60 *μ*M putrescine, 30 nM selenium on poly-d-lysine coated plates. Cultures were used for experimentation 7 days after plating. Primary rat sympathetic culture was prepared as described earlier from superior cervical ganglia of neonatal rat pups.^46^ NGF deprivation was performed as described previously.^47^ In brief, the fully differentiated PC12 cells or sympathetic neurons were washed with NGF-free medium and anti-NGF antibody (1 : 100; 5 *μ*g/ml) was added. Control cells were washed with serum-free medium and maintained in NGF containing medium. To study the effect of Cdc25 inhibitors on differentiated PC12 cells or sympathetic neurons, cultures were subjected to NGF deprivation overnight in the presence and absence of inhibitors. The cells were then processed for cell survival or western blotting.

### Preparation of amyloid

A*β*_(1–42)_ and A*β*_(42–1)_ were prepared as described earlier.^48^ In brief, HPLC-purified A*β*_(1–42)_ and A*β*_(42–1)_ were purchased from American Peptide. Lyophilized A*β*_(1–42)_ or A*β*_(42–1)_ was reconstituted in 100% 1,1,1,3,3,3 hexafluoro-2-propanol (HFIP) at 1 mM, HFIP was removed by evaporation in a Speed Vac (Hamburg, Germany), then the material was resuspended at 5 mM in anhydrous DMSO. This stock was then stored in −80 °C. For use, the stock was diluted with PBS to a final concentration of 400 *μ*M and SDS was added to a final concentration of 0.2%. The resulting solution was incubated at 37 °C for 18–24 h and then diluted with PBS to a final concentration of 100 *μ*M and incubated at 37 °C for 18–24 h before use.

### Assessment of cell survival

The intact nuclear counting assay was performed as described earlier^23^ by using a detergent containing buffer that dissolves the cell contents, but leaves the nuclei intact. The intact nuclei were then counted using a hemocytometer. The number of live cells was expressed as the percentage of the total cell population.

### RNA isolation and PCR

Total RNA was extracted using TRI reagent (Sigma) as described earlier.^49^ cDNA was prepared using the cDNA synthesis kit from Applied Biosystems (Waltham, MA, USA) following the manufacturer’s protocol. For each reaction 100 *μ*g of total RNA was used. The cDNA synthesis was done using an oligo dT primer. The cDNA were used for semiquantitative and quantitative PCR using a BioRad (Hercules, CA, USA) and ABI step one plus machines, respectively, using specific primers. Primer sequences for Cdc25A were 5′-CAGCTTCCACACCAGTCTCT-3′ and 5′-TTGACTGCCGATACCCATAT-3′. The primers for *α*-tubulin were 5′-ATGAGGCCATCTATGACATC-3′ and 5′-TCCACAAACTGGATGGTAC-3′. For semiquantitative PCR, products were analyzed on a 1.5% agarose gel and visualized by staining with ethidium bromide. Quantitative PCR (qRT-PCR) was performed using ABI SYBR green master mix and ABI Step one plus machine following the manufacturer’s protocol. For miRNA quantification, the RNA was isolated as described above and analyzed for microRNA levels using Taqman assay. The miRNA probes were purchased from Ambion (Life technologies).

### Western blotting

The cells were pelleted and lysed in lysis buffer (10 mM Tris (pH 7.4), 150 mM NaCl, 1% Triton-X 100, 1 mM EDTA, 1 mM EGTA, 0.2 mM activated orthovanadate) on ice for 20 min. The lysates were centrifuged at 12 000 r.p.m. for 10 min. The supernatant was collected and used for western blotting analysis. Fifty micrograms of the proteins were resolved by SDS-PAGE. After electrophoresis the proteins were transferred from the gel to PVDF (Hybond: GE Healthcare) membranes. The membranes were blocked with 5% non-fat dry milk (NFDM) (Biorad) in 1× TBST for 1 h at room temperature on a shaker. Then primary antibody was added and incubated overnight at 4 °C (5% BSA in TBST was used as primary antibody diluent) on a rotating shaker. The membranes were washed three times for 5 min with TBST and the HRP-conjugated secondary antibody was added to the appropriate dilution and the membranes were incubated for 1 h on a shaker. The membranes were washed with TBST three times for 5 min and chemiluminescence assays were carried out using the Milipore classic luminol reagent according to the manufacturer’s protocol. Signals were detected on X-ray films (Kodak, Windsor, CO, USA).

### Immunocytochemistry

Immunocytochemical staining of cultured cells was carried out following previously described procedures.^18,50^ Briefly, neuronal cells were grown on glass coverslips, fixed with 4% PFA, blocked with 3% goat serum in PBST for 1–2 h and then incubated overnight with primary antibody in blocking solution. After washing, the coverslips were incubated with secondary antibody for 1 h and then incubated with Hoechst dye for nuclear staining and imaged under fluorescence microscopy (Leica, Wetzlar, Germany) with a digital spot camera.

### Phosphatase assay

The Cdc25A phosphatase assay was performed using the *p*-nitrophenyl phosphate liquid substrate system (Sigma Aldrich, St Louis, MO, USA) according to the manufacturer’s protocol. Briefly, the protein lysates were incubated with anti-Cdc25A antibody for overnight at 4 °C under shaking. The following day 25 *μ*l of protein A agarose was added and incubated for 2 h. Then the agarose-conjugated Cdc25A were pelleted by centrifugation. The immunoprecipitated Cdc25A was incubated with 200 *μ*l of *p*-nitrophenyl phosphate liquid substrate for 30 min at room temperature in the dark. The absorbance of the solution was then read at 405 nm in an ELISA reader.

### Preparation of shRNA

shRNAs were prepared in the pSIREN vector by using the BD Biosciences knockout RNAi system, according to the manufacturer’s instructions on the basis of the following sequence 5′- gccattctgattctctaga-3′.

### Transfection and gene silencing

DNA was prepared with the Plasmid Maxi kit (Qiagen, Waltham, MA, USA). For the survival assay, neuronal cells were transfected with 0.5 *μ*g of the specific plasmid using Lipofectamine 2000 following the manufacturer’s protocol. Six hour later, medium containing Lipofectamine 2000 and DNA was replaced with fresh complete medium. Experimental assays were performed 48 h post transfection. The numbers of surviving-transfected (green) cells per well were assessed just after treatment and at different time intervals as indicated.

### Survival assay

Primary cortical neurons (5 DIV) and sympathetic neurons (5 DIV) were transfected with the indicated shRNA constructs. After 48 h of transfection, the neurons were exposed to A*β*_(1–42)_ or anti-NGF, respectively. The numbers of transfected neurons (green) were counted immediately (0 h) and after 24 and 48 h of treatment. Neuronal PC12 cells were cultured in 24-well plates. After 3 days, cells were transfected with the indicated shRNAs using Lipofectamine 2000. Forty-eight  hours post transfection, cells were washed with NGF-free medium twice and anti-NGF antibody (1 : 100) was added. The numbers of surviving-transfected cells were counted under fluorescent microscopy.

### Oligomeric A*β* infusion in animals

Infusion of A*β*_(1–42)_ was performed as described earlier.^23^ Male Sprague-Dawley rats (300–380 g) were anesthetized by injecting a mixture of xylazine–ketamine and placing them on a stereotaxic frame. Injection was carried out by using a 27-gauge Hamilton syringe. A volume of 5 *μ*l of 100 mM A*β*_1–42_ in PBS was infused in the right cerebral cortex at stereotaxic co-ordinates from bregma: AP: −4.1, L: 2.5, DV: 1.3 mm, according to a previous report.^51^ An equal volume of A*β*_42–1_ in PBS was injected in control animals. Animals were killed 21 days after injection. The brains were dissected out, following cardiac perfusion, and fixed in 4% PFA for 24 h. They were then incubated in a 30% sucrose solution for another 24 h before proceeding with cryosectioning (Cryotome; Thermo).

### Immunohistochemistry of brain slices

Twenty micrometer brain cryosections from A*β*-infused or PBS-infused rats and wild-type or transgenic mice were blocked with 5% goat serum in PBS containing 0.3% Triton-X 100 for 1 h at room temperature. Sections were incubated in primary antibody in a blocking solution overnight at 4 °C, washed thrice with PBS and incubated with a fluorescence-tagged secondary antibody for 2 h at room temperature. Following three washes with PBS and Hoechst dye nuclear staining, the sections were mounted and observed under fluorescence and confocal microscopy (Leica).

### Statistics

The experimental results are reported as mean±S.E.M/S.D. Student’s *t*-test was performed as unpaired, two-tailed sets of arrays to evaluate the significance of differences between the means of experimental and control groups and the results are presented as *P*-values. One-way ANOVA was performed for data sets of more than two groups.

## Abbreviations

Aβ: β-amyloid
AD: Alzheimer’s disease
Cdc25A: cell division cycle 25A
CDK4: Cyclin dependent kinase 4
FoxO: forkhead box, class O
miR21: micro RNA 21
NGF: nerve growth factor
PC12: pheochromocytoma cells
Rb: retinoblastoma
RNAi: RNA interference
shRNA: small hairpin RNA
